# The 3rd Educational Course of the European Burns Association (EBA)

**DOI:** 10.3390/ebj5040032

**Published:** 2024-10-21

**Authors:** Nadia Depetris, Alette E. E. de Jong, Clemens Schiestl, Frank Siemers, Jill Meirte, Jyrki Vuola, Luís Cabral, Paul Van Zuijlen, Stian Almeland

**Affiliations:** 1 Burn Center, CTO Hospital, Città della Salute e della Scienza, 10126 Turin, Italy; 2 Burn Center, Rode Kruis Ziekenhuis, 1942 LE Beverwijk, The Netherlands; aeedejong@rkz.nl; 3 Department of Surgery, University Children’s Hospital Zurich, 8032 Zürich, Switzerland; clemens.schiestl@kispi.uzh.ch; 4 Department of Plastic, Hand Surgery and Burn Care, BG Klinikum Bergmannstrost, 06112 Halle, Germany; frank.siemers@bergmannstrost.de; 5 Research Group MOVANT (Movement Antwerp), Department of Rehabilitation Sciences and Physiotherapy, University of Antwerp, 2000 Antwerp, Belgium; jill.meirte@uantwerpen.be; 6 OSCARE, Organisation for Burns, Scar Aftercare and Research, 2170 Antwerp, Belgium; 7 Helsinki Burn Centre, Department of Plastic Surgery, Jorvi Hospital, Helsinki University Hospital, University of Helsinki, Karvasmäentie 8, 02740 Espoo, Finland; jvuola@icloud.com; 8 Department of Plastic Surgery and Burns Unit, Coimbra University Hospital Centre (CHUC), 3000-075 Coimbra, Portugal; jlacabral@gmail.com; 9 Department of Plastic Reconstructive and Hand Surgery, Movement Sciences (AMS) Institute, Amsterdam UMC, Location VUmc, 1081 HZ Amsterdam, The Netherlands; paulvanzuijlen@me.com; 10 Burn Center and Department of Plastic and Reconstructive Surgery, Red Cross Hospital, 1942 LE Beverwijk, The Netherlands; 11 Association of Dutch Burn Centers (ADBC), 1941 AJ Beverwijk, The Netherlands; 12 Paediatric Surgical Center, Emma Children’s Hospital, Amsterdam UMC, 1105 AZ Amsterdam, The Netherlands; 13 Department of Plastic, Hand and Reconstructive Surgery, Norwegian National Burn Center, Haukeland University Hospital, NO-5021 Bergen, Norway; stian.almeland@gmail.com; 14 Department of Clinical Medicine, University of Bergen, NO-5021 Bergen, Norway

**Keywords:** burns, burn care, burn center

## Abstract

Abstracts of the plenary sessions, workshops, and poster presentations of the 3rd EBA Educational Course in Porto, Portugal, 17–18 October 2024.

## 1. Introduction

We are pleased to present the official report of the 3rd EBA Educational Course, focused on the critical topic of “Burn Wounds and Infections”. The high prevalence of infections in severe burns, coupled with their complex management, underscores the importance of a multidisciplinary approach.

Held in Porto, Portugal, on 17 and 18 October 2024, the course offers insights from renowned burn care experts, hands-on learning, and the latest research. Attendees have the opportunity to delve into burn wound management, participate in interactive workshops, and explore cutting-edge research through poster presentations.

The 3rd EBA Educational Course provides a valuable platform for healthcare professionals to expand their knowledge, network, and contribute to the advancement of burn care.

This comprehensive report highlights the enriching sessions, interactive workshops, and posters presented during the event.

## 2. Acknowledgments

Thanks are due to all the EBA Committees cooperating for this meeting. The assistance of all the staff members of Congress Care and the Editorial Office of the European Burns Journal in preparing this Educational Course is gratefully recognized. All the industries and companies which supported this event are also appreciatively acknowledged. Moreover, deep gratitude must be shown to all the professionals, researchers, burn survivors’ associations, and family members who actively and incessantly work to improve burn care.

## 3. Plenary Sessions and Workshops

*PS.001*

Thursday 17 October 09:30 a.m.–11:00 a.m.

Title

- Moderators: Luis Cabral (Portugal) and Clemens Schiestl (Switzerland)
- Speakers: Laura Pompermaier (Sweden), Nadia Depetris (Italy), and Dominique Navet Potokar (France)

Burns remain a significant global health threat, with a high incidence and mortality rate. This session will explore the epidemiology of burns, focusing on infections as a common complication. The session will further delve into the intricate relationship between burns, infections, and septic shock, underscoring the critical role of timely and effective infection management in preventing these devastating sequelae. Finally, the importance of a multidisciplinary approach for the prevention, diagnosis, and treatment of burn-related infections and sepsis will be presented.

*PS.002*

Thursday 17 October 11:30 a.m.–12:30 p.m.

Early excision vs. late excision: pros and cons debate

- Moderators: Thomas Leclerc (France) and Valentina Strbac (Croatia)
- Speakers: Jyrki Vuola (Finland) and Stian Almeland (Norway)

This debate will explore the scientific evidence for early and late excision in burns, addressing misconceptions and organizational challenges too. The participants will discuss the benefits and drawbacks of each approach, considering factors such as wound healing, infection rates, and resource utilization. The aim is to inform clinical decision making and identify best practices in burn care.

*PS.003*

Thursday 17 October 16:00 p.m.–17:30 p.m.

Pharmacological approach to infections in burns

- Moderators: José Ramon Martinez Mendez (Spain) and Mamta Shah (United Kingdom)
- Speakers: **Paul van Zuijlen (The Netherlands)**, **Marisa Caetano (Portugal)**, and **Thomas Leclerc (France)**

This session will explore various approaches to managing infections in burns, including topical treatments, systemic pharmacology, and the emerging field of phage therapy. Experts will discuss the benefits, limitations, and challenges associated with each approach, providing insights into the current best practices and future directions in burn care. The session will also address the importance of a multidisciplinary approach to managing infections in burns, involving a combination of pharmacological interventions and supportive care.

*PS.004*

Friday 18 October 8 a.m.–9 a.m.

The delayed admission—a problem with many aspects

- Moderators: Nadia Depetris (Italy) and Frank Siemers (Germany)
- Speakers: Clemens Schiestl (Switzerland), Stian Almeland (Norway), Wera Wendenburg (Germany), and Christine Rosch (Switzerland)

Nearly all burn centers in Europe have admitted in the last few years burned patients from abroad, mainly from countries with low-level burn care or war zones. Often, two to three weeks after the trauma with any surgical treatment besides wound dressings are colonized and/or infected by multi-resistant bacteria. These patients represent a challenge in the view of surgical treatment strategies, infection management, nursing, psychosocial, and ethical problems. In a “One-Day Topic” interactive format, we will present and discuss all the different aspects in the plenum and in small workshops. The four interactive workshops will be facilitated by international multidisciplinary burn experts. The conclusions will be presented in a plenary session, allowing a further and deeper joint exploration of the theme.

*W001*

Friday 18 October 9 a.m.–12 p.m.

Ethics

Laura Pompermaier (Sweden), Stian Almeland (Norway), Nadia Depetris (Italy), Dominique Navet Potokar (France), Frank Siemers (Germany), and Romana Merza (Ukraine).

*W002*

Friday 18 October 9 a.m.–12 p.m.

Surgery

Clemens Schiestl (Switzerland), Tom Potokar (France), Jyrki Vuola (Finland), Lars-Peter Kamolz (Austria), and Olga Kovalenko (Ukraine).

*W003*

Friday 18 October 9 a.m.–12 p.m.

Infection control and MDR/XDR pathogens

Thomas Leclerc (France), Valentina Strbac (Croatia), Lesia Strilka (Ukraine), Luis Cabral (Portugal), Marisa Caetano (Portugal), and Paul Van Zuijlen (The Netherlands).

*W004*

Friday 18 October 9 a.m.–12 p.m.

Nursing and psychosocial aspects

Alette de Jong (The Netherlands), Sigrid Brokke (Norway), Jill Meirte (Belgium), Halyna Sayan (Ukraine), and José Ramon Martinez Mendez (Spain).

*PS.005*

Friday 18 October 13 p.m.–15 p.m.

Plenary presentation and discussion

- Moderators: Nadia Depetris (Italy) and Clemens Schiestl (Switzerland)

The conclusions of the workshops will be presented in a plenary session, allowing a further and deeper joint exploration of the theme “The delayed admission—a problem with many aspects”.

*PS.006*

Friday 18 October 16 p.m.–17 p.m.

Is it possible to prevent infections in severe burns? Round table

- Moderators: Paul Van Zuijlen (The Netherlands) and Dominique Navet Potokar (France)

A roundtable discussion will explore the strategies to prevent infections in burn patients. Experts from various fields will discuss the latest advancements in burn care, infection control, and emerging technologies. The goal is to identify effective methods for improving patient outcomes and reducing the burden of burn-related infections.

## 4. Poster Presentations

*Poster session 1, 17 October 2024, 11:00–11:30*

*P01*

Burn injury and chronic pain

**Dr. Rui Calvinho Almeida** ^1^, Pedro Franco Santos ^1^, Renato Borges ^1^, Gonçalo Ferreira ^1^, Dmitry Shelepenko ^1^, José Miguel Azevedo ^1^, Inês Catalão ^1^, Miguel Sítima ^1^, Susana Pinheiro ^1^, MD, and PhD Luís Cabral ^1^.

^1^ Coimbra Hospital Center, Coimbra, Portugal.

Aim:

To evaluate the long-term consequences of burns, particularly in the development of chronic pain; identify the respective predictive factors; and assess their socioeconomic impact.

Methods:

A retrospective observational study at the Coimbra’s Burns Unit from 1 December 2019, to 31 May 2023. Inclusion criteria: 2nd- and/or 3rd-degree burns, TBSA > 10%, <65 years old, and interview based on the Structured Interview Protocol, Douleur Neuropathique (DN4), and Socioeconomic Deprivation Index (SEDI) questionnaires (n = 74). The study focuses on variables such as chronic pain and its characteristics, the duration of medical leave, productivity upon returning to work, the development of psychiatric conditions, and the impact on sleep and social life.

Results:

Thirty patients (40.5%) developed chronic pain after the burn, with the majority being neuropathic in nature (33.8%) and localized in the upper limb (56.7%). The only predictive factor identified was age, with an OR of 0.95 (95% CI 0.907–0.994) and *p* = 0.027, with a decrease in risk as age increases.

A high prevalence of sleep disturbances (28.4%), impact on social life (35.1%), and psychiatric disorders (20.5%) such as depression, anxiety, and PTSD was observed. Interestingly, longer medical leave correlates with higher socioeconomic classes (F = 6.376, *p* = 0.035), while higher productivity correlates with lower socioeconomic classes (F = 16.757, *p* < 0.001).

Conclusions:

Advanced age is associated with a lower risk of developing chronic pain. The impact of the burn should be framed within the patient’s biopsychosocial context, and more rigorous follow-up after hospital discharge may help prevent and treat the most common complications.

*P27*

Treatment of an extensive burn with pla-membrane

**Dr. Ina Nietzschmann** ^1^

^1^ BG-Klinik Halle, Leipzig, Germany

Objective:

We took over a 39-year-old Ukrainian soldier after a gas cylinder explosion at the front with a burn of 80% body surface 2a–b grade.

Method:

We would like to present an individual case that is characterized by a protracted course and a special spectrum of germs.

Findings:

We treated a patient who was referred to us 4 weeks after the accident and whose initial treatment had taken place in Ukraine. On admission, the wound was found to be colonized with multi-resistant germs. After extensive debridement and the subsequent application of PLA-membrane as well as a hygiene concept established in our burn center, all burn wounds were healed and the patient was discharged for rehabilitation.

Conclusions:

Patients who are secondarily referred to us with burn wounds colonized with multi-resistant germs pose a major challenge in terms of therapy and hygiene management as well as personnel costs.

*P29*

Using a Digital Platform and a Virtual Ward Model to Integrate Care for Burns Patients in Non-Specialist Areas

**Mrs. Alice Eastland Prosser**, **Mamie O’Reilly** ^1^

^1^ Chelsea & Westminster Hospital, London, United Kingdom

Aim:

Utilizing digital platforms and virtual wards to enhance burn care.

Methods:

Our methodology involved reviewing data from patients referred to the burn unit outreach service between January and March 2024. We collected information on age, burn location, TBSA, depth, management, time to heal, and complications.

Results:

A total of 30 patients were identified (12 female; 18 male).

Conclusions:

Integrating a digital platform with a ward model for burn outreach patients optimizes care and resource allocation, enhancing efficiency and extending our reach within the patient population while maintaining high standards.

This service represents a significant leap forward in our practice. The platform’s remote monitoring capabilities swiftly detect complications like burn wound infections, enabling timely interventions that enhance patient outcomes and trim costs, notably in transport fees and hospital stays.

Additionally, virtual consultations reduce outreach mileage costs, prioritizing face-to-face care for other burns patients.

By utilizing the platform for smaller burns, resources can be allocated to larger ICU-level cases, where our expertise is crucial.

This redistribution not only relieves pressure on our burn unit but also offers focused attention to other burn patients in non-specialist areas.

In essence, the digital platform is more than a tool; it is a vital asset streamlining workflow, optimizing resource allocation, and elevating care standards for burns patients, marking a significant stride forward in efficiency, effectiveness, and excellence in our burns outreach service.

*P30*

Enzymatic debridement—why? when? where? how? Comparative study in a single burn center

**Mihaela Pertea** ^1,2^, Malek Benamor ^2^, Oxana-Madalina Grosu ^1,2^, Stefana Luca ^1,2^

^1^ Grigore T. Popa University of Medicine and Pharmacy, Iasi, Romania, ^2^ Department of Plastic Surgery and Reconstructive Microsurgery and Burn Unit, Sf Spiridon, Emergency County Hospital, Iasi, Romania

Aim:

This study aims to highlight the indications and results of applying a mixture of proteolytic enzymes with high bromelain content for the treatment of deep burns in a single center.

Methods:

The study included two groups (one group of 15 patients and another of 30 patients) with burn surfaces between 10 and 45% TBSA and depths of IIB and III. The first group was subject to early excision and skin grafting; for the second group, enzymatic debridement was used (including the face). We recorded the effectiveness of enzymatic debridement, the healing time, the need for grafting after debridement, the aesthetic aspect of the scars, and patient satisfaction. Both the classic skin graft technique and the Meek micrografting technique were used.

Results:

The enzymatic debridement efficiency was between 85 and 92%. In 8 cases out of 30, skin grafts were necessary. Spontaneous epithelization was noticed in the other 22 cases. The patients recorded less pain, less bleeding, and less damage to surrounding healthy tissues than the group that underwent surgical debridement.

Conclusions:

The introduction of enzymatic debridement as an alternative to surgical debridement for IIB- and III-degree burns has changed standard burn care. This method has the additional benefit of selectivity—the removal of non-viable tissue and preservation of viable tissue, reducing the number of surgical interventions, infection rates, and days of hospitalization with a very good aesthetic result.

*P31*

Reopening the burn ICU after Acinetobacter outbreak—a multi-professional challenge

**Christine Rosch** ^1^

^1^ Universitätsspital Zürich, Zürich, Switzerland

Objective:

Avoiding a new outbreak with adapted processes and a common attitude for all professional groups.

Methods:

During the outbreak, various procedures to eliminate the germ were carried out over a period of months without success.

Weekly multi-professional meetings between representatives of all specialist disciplines and the hospital hygiene department.

After several setbacks, the remaining unaffected patients were moved to another intensive care unit. Clearing out and sterilizing the entire burns intensive care unit.

Exchange of experience with burn centers in Germany. Visit to a burn center by a multi-professional delegation.

The adaptation of work processes in all the specialist disciplines and the joint exchange of information.

Results:

A new start in a clean burn intensive care unit with adapted processes to prevent a new outbreak.

New shared awareness of the importance of multi-professional responsibility for hygiene around the patient pathway.

Adapted concepts for the different patient locations.

New and more rigorous concepts to prevent the contamination of storage rooms even in the event of a new outbreak.

New and more rigorous disinfection strategies for equipment and rooms.

Conclusions:

Long-lasting rollercoaster ride for the entire multi-professional team.

Extremely high costs for the disposal of all the materials in the ICU and for the sterilization of equipment and rooms.

Difficulties in finding our own way of dealing with this situation.

Finally, a common understanding of the adapted processes.

*P32*

Fish skin for the treatment of deep dermal wounds

**Prof. Dr. Jennifer Schiefer** ^1^

^1^ Cologne Merheim Medical Center/University of Witten/Herdecke, Cologne, Germany

Aim:

It is known that long-term scarring from burns is significantly reduced through the use of enzymatic debridement; nevertheless, up to date, many centers use skin grafts for deep dermal burns. Kerecis Omega3 Wound^®^, derived from fish skin, could be an alternative to this.

Methods:

Therefore, fish skin was applied after the enzymatic debridement of the deep dermal burns of the hand and foot and evaluated closely.

Results:

In 2022 and 2023, 18 patients between the ages of 18 and 52 years with deep dermal burns were treated. The mean healing time was 16 days. The management of dressing changes, the assessment of the progress of wound healing, and adjusting the level of moisture in the dressings were obstacles, which could only be overcome by the appropriate experience of the burn surgeon. In particular, adjusting the degree of moisture had a major influence on the progress of wound healing. Depending on these parameters, there were very different healing processes of the fish skin, from early dissolution into the so-called “active slough”, to the drying out of the wound dressing to form a crust and remaining there for several weeks.

Conclusions:

In all 18 patients, however, the treatment led to spontaneous wound healing and satisfactory results without the need for skin grafting or other surgical procedures. Kerecis Omega3 Wound^®^ is safe to use and suitable for the treatment of deep dermal burn wounds following enzymatic debridement and should be compared to other dressings in the future.

*P33*

Mortality analysis in a major burn center 5-year follow-up

**Dr. Dmitry Shelepenko** ^1^, Dr. Gonçalo Tomé ^1^, Dr. José Miguel Azevedo ^1^, Dr. Inês Catalão ^1^, Dr. Miguel Sítima ^1^, Dr. Rui Almeida ^1^, Dr. Miguel Vaz ^1^, Prof. Luís Cabral ^1^

^1^ Coimbra Hospital and University Centre (CHUC), Coimbra, Portugal

Aim:

Death is the reality for approximately 10–20% of patients in burn units in developed countries (1). The aim of this study was to identify the typical characteristics of this population and factors that may alter their clinical outcomes.

Methods:

The authors analyzed the medical records of patients admitted to a major burns center between 1 January 2017, and 1 January 2022. Deceased patients were compared to the general burnt population and to survivors. The comparison factors used were the cause and location of the accident, total body surface area (TBSA), body parts involved, length of hospital stay, and time of year that death occurred.

Results:

During the sampling period, 683 patients were admitted (284 women and 399 men), of which 70 had died (mortality rate ~10%) and their median age was 69 years (vs 60 years in the general group). The main cause of burns in the deceased patients was fire (66%) usually at home (66%), and approximately 63% had more than 20% of their body burned. The highest mortality periods were at 3–4 days of hospital stay and uniformly in the following 3 weeks after the accident.

Conclusions:

It is not news that deceased patients often share common characteristics, such as having more than 20% of their body surface burned. However, there are other less obvious commonalities, such as the high average length of hospital stay (27 vs. 21 days in the population that survived). This temporal relationship may justify an extra investment in some severely burned patients or help to adopt palliative measures earlier.

*P34*

Enzymatic debridement of burn wounds with bromelain: 4 years of experience

**Dr. Miguel Sítima** ^1^, Gonçalo Tomé ^1^, Dmitry Shelepenko ^1^, José Miguel Azevedo ^1^, Inês Catalão ^1^, Rui Almeida ^1^, Miguel Vaz ^1^, Carla Diogo ^1^, Luís Cabral ^1^

Centro Hospitalar E Universitário De Coimbra, Coimbra, Portugal

Aim:

To describe our protocol, work, and experience with the bromelain debridement of burn wounds in the last 4 years.

Methods:

We reviewed the patients treated with bromelain in Coimbra’s Burn Unit in the last 4 years (n = 12) and analyzed a range of variables, specifically age, burn depth, etiology, application site, total burn surface area (TBSA), length of stay (LOS), associated injuries, and need for surgical debridement/grafting.

Results:

Our protocol, in line with the European guidelines, gives preference to an early bromelain application, until 72 h after injury, in mixed pattern deep burns. Since 2021, we performed 12 enzymatic debridements on a patient sample with a median age of 57 years old, all of whom suffered from deep partial- to full-thickness burn injuries. The most frequent causes were gas explosions and clothing/gasoline ignition. TBSA ranged from 3 to 80% and most debridements were performed either on the upper or lower limbs (n = 5). All patients were treated 12–72 h after injury. LOS ranged from 5 to 79 days and, in most cases (n = 6), there was no need for additional surgical debridement or skin grafting.

Conclusions:

Despite its limited indications and high financial cost, enzymatic debridement with bromelain remains a useful and effective resource in the treatment of deep burn wounds, with the potential of avoiding the need for surgical debridement in many cases.

*P36*

New multimodal approach in treatment of deep partial-thickness and full-thickness burns after enzymatic debridement

**Panche Taskov** ^1,2^, Daniela Monica Corodati ^2^, Zorin Petrisor Crainiceanu ^1,2^

^1^ University Of Medicine And Pharmacy Victor Babes Timisoara, Timisoara, Romania, ^2^ County Hospital Pius Brinzeu—Clinic for Plastic and Reconstructive Surgery, Timisoara, Romania

Aim:

A novel concept consisting of the selective enzymatic debridement of deep partial-thickness and full-thickness burns in combination with tissue micrografting technique using autologous micrografts, PRP, and “smart” topics and dressings are used in order to avoid SOC treatment.

Method:

Selective enzymatic debridement was developed for the removal of thermic burn eschars which, at the same time, preserves the viable tissue, mainly the dermis.

Tissue micrografting is useful when there are insufficient donor sites able to provide the required amount of skin grafts because only a small skin biopsy is necessary. The micrograft suspension obtained by mechanical disaggregation can be injected directly or in combination with collagen scaffolds.

Smart hyaluronic acid-based topics and dressings generate a microenvironment that supports the healing process.

PRP promotes healing by accelerating cell migration, and proliferation of fibroblasts and participates in hemostasis and coagulation.

Biotechnology microbial-derived NanoCellulose wound dressing reduces the intradermal damage of the skin and creates a supportive moist environment for the wound.

Results:

All the areas treated with “MA” did not require additional surgery and coverage. In ~3 weeks the lesions were completely epithelized.

The quality of the scars was evaluated according to the Vancouver scale.

The elasticity, quality, and aesthetic aspect of the scars are superior in comparison with SOC scar quality.

Conclusions:

Burns need a new multimodal approach.

This novel regenerative technique has shown promising results in the burn healing process, reduces hospitalization and healing time, and improves quality of life.

Keywords:

enzymatic debridement; tissue micrografting; biotechnology microbial-derived nanocellulose

*P37*

Comprehensive Management of Delayed Presentation Full-Thickness Facial and Hand Burns: A Case Study

**Ms. Theodora Tsoliaridou** ^1^ T. Tsoliaridou ^1^, V. Kastaboulidou ^1^, A. Emmanouilidis ^1^, C. Tsakiri ^1^, E. Nikolaidou ^1^, K. Makarona ^1^, Z. Tzimorota ^1^, S. Papadopoulou ^1^

^1^ G. Papanikolaou: Hospital, Thessaloniki, Greece, Thessaloniki, Greece

Aim:

To present the challenges and outcomes of the management of an elderly patient with seriously delayed presentation of full-thickness burns to the face and hands.

Methods:

An 83-year-old male patient with about 10% total body surface area burns presented to our outpatient clinic 35 days post-injury with eschars on the face and infected defects on both hands. The initial management included surgical debridement plus Versajet, STSG on the face and left hand, and VAC therapy for the right hand. At a second stage, a reverse radial forearm flap was used to reconstruct the defect of the dorsum of the right hand a week later.

Results:

Post-operatively, the patient was managed and intubated in the BICU for 24 h. After weaning, he still stayed for a week in BICU due to delirium and was then transferred to the ward. Challenges included the patient’s initial treatment refusal and communication barriers due to pain and psychological distress, necessitating psychiatric intervention. Despite these, the patient’s wounds healed after 25 days.

Conclusions: This case highlights the essential role of continuous, specialized nursing care in managing severe burns in elderly patients. Nursing care was crucial for monitoring surgical interventions, addressing psychological and rehabilitative needs, and facilitating recovery and compliance with treatment protocols.

*P38*

The antimicrobial efficacy of topically applied mafenide acetate, citric acid, and wound irrigation solutions Lavanox and Prontosan against Pseudomonas aeruginosa

**Dr. Maria von Kohout** ^1^, Prof. Dr. med. Paul Christian Fuchs ^1^, PD. Dr. rer. nat. Christian Oplaender ^1^, Prof. Dr. med. Jennifer Lynn Schiefer ^1^

^1^ Burn Center Cologne-Merheim Hospital, Witten/Herdecke University, Cologne, Germany

Abstract:

Since burn wound infections caused by Pseudomonas aeruginosa (PA) lead to major complications and sepsis, this study evaluates the antimicrobial efficacy of the wound irrigation solutions Prontosan (PRT), Lavanox (LAV), citric acid (CA), and mafenide acetate (MA) using microbiology assays and an ex vivo skin wound model. In suspension assays, all the solutions showed significant reductions in bacteria number (log10 reduction: CA 5.77; LAV 4.91; PRT 4.74; MA 1.23). The biofilm assay revealed that PRT and LAV reduced the biofilm formation by ~25% after a 15 min treatment, while PRT was most effective after a 24 h treatment (~68%). All the treatments (24 h) reduced the number of PA found in biofilms (1.36–1.65 log10 reduction), whereas after a 15 min treatment, CA and LAV were the most effective (log10 reductions ~2.5). In the skin wound model, PRT and LAV provided the highest bacterial reduction after a 15 min treatment (log10 reduction 1.8–1.9), while MA was more effective after a 22 h treatment (log10 reduction 3.6). The results demonstrated the antimicrobial efficacy of all the solutions against PA. Further investigation is needed to explore the potential clinical applications of a combination or alternating use of these solutions for infection prophylaxis and the treatment of wound infections caused by PA.

*P39*

Assessing First Aid Knowledge for Burns Among the Maltese Population: Identifying Gaps and Informing Public Health Strategies

**Christine Vella** ^Mater Dei Hospital^, Juanita Parnis ^Mater Dei Hospital^, Dr. David Borg

Burn injuries are a significant public health concern due to their potential for causing severe physical damage, long-term disability, and even death. Prompt and effective first aid can mitigate the severity of burn injuries, reduce complications, and improve overall outcomes. However, the knowledge of appropriate first aid measures for burns varies widely among the general public, often leading to improper treatment. This study aims to evaluate the level of first aid knowledge for burn injuries among the Maltese population and identify specific knowledge gaps that require targeted educational interventions.

A cross-sectional survey design was employed, utilizing a structured questionnaire comprising 15 scenarios to assess participants’ knowledge of appropriate first aid measures for various burn types, including thermal, electrical, and chemical burns. The questionnaire was provided in both Maltese and English to ensure comprehensive understanding. The participants were recruited through convenience sampling from public areas, ensuring a diverse representation of age groups and socioeconomic backgrounds. Informed consent was obtained, and data protection regulations were strictly adhered to.

The results indicated a mixed level of awareness among the participants, with significant gaps and misconceptions. While 50% of the participants correctly identified the appropriate response for a child who pulls boiling water onto themselves, only 44% knew the correct response for an acid splash on the face. Notably, 100% incorrectly believed that burns always lead to scarring. However, 92% correctly identified that ice should not be used directly on burnt skin, and 87% knew that cold running water is the appropriate method for cooling a burn.

These findings highlight the need for targeted educational interventions to improve first aid knowledge among the Maltese population. By addressing these knowledge gaps through public health strategies and educational programs, the management of burn injuries can be significantly improved, ultimately enhancing the health outcomes and reducing the burden on healthcare systems.

*Poster session 2, 17 October 2024, 12:30–13:00*

*P02*

An analysis of pain management and mortality risk assessment in Coimbra’s Burn Unit

**Dr. Rui Calvinho Almeida** ^1^, Pedro Franco Santos ^1^, Renato Borges ^1^, Gonçalo Ferreira ^1^, Dmitry Shelepenko ^1^, José Miguel Azevedo ^1^, Inês Catalão ^1^, Miguel Sítima ^1^, Susana Pinheiro ^1^, MD, PhD Luís Cabral ^1^

^1^ Coimbra Hospital Center, Coimbra, Portugal

Aim:

To characterize the population of Coimbra’s Burn Unit, comparing pain management with different analgesic protocols and identifying factors associated with increased mortality.

Methods

A retrospective observational study at Coimbra’s Burns Unit from 1 December 2019, to 31 May 2023, including all the patients admitted with burn diagnosis (n = 452). The daily monitoring of the patients’ pain intensity was evaluated through a visual numeric pain scale and classified as light (≤3), moderate (4–6), and intense (≥7) pain. The pain was also classified as well controlled, partially controlled, or poorly controlled according to the overall pain scores. More than 10 types of analgesia protocols were implemented.

Results:

A small correlation between TBSA, number of surgeries (r = 0.225, *p* < 0.001), balneotherapies (r = 0.204, *p* < 0.001), and duration of hospital stay (r = 0.289, *p* < 0.001) was verified. Through a linear regression between TBSA and duration of hospital stay, we also identified that for each 2.86% increase in TBSA, the duration of stay would increase by 1 day. No predictive factors for pain were identified regarding adequate pain management, which emphasizes the need for a personalized approach.

Age and TBSA proved to be mortality predictors, both included in the Baux Score. However, inhalation injury was not a significant mortality predictor, despite the survival time being lower in this group. The number of hydrotherapy sessions was with a lower mortality.

Conclusions:

No difference was identified in terms of the analgesic regimens used. An increase in age, TBSA, and the number of days on IMV were associated with an increase in mortality, whereas balneotherapy sessions were associated with a reduction in mortality.

*P03*

Management of thermal burns in neonates in a tertiary center—a 5-year retrospective study

**Prof. Maya Argirova** ^1^, Management of thermal burns in neonates in a tertiary center—a 5-year retrospective study; Yolanda Zayakova ^2^, Management of thermal burns in neonates in a tertiary center—a 5-year retrospective study Anastasiya Viktorova^1^

^1^ Umhatem “n.i. Pirogov”, Sofia, Bulgaria, ^2^ Department of Burns and Plastic Surgery, MBAL, Varna, Bulgaria

The aim of the study is to analyze retrospectively the magnitude of injury, local wound management, role of surgery, and outcome in neonatal burns admitted to two Burns Centers.

Material and Methods: Between 2019 and 2023, nineteen neonates, aged less than 28 days with burns of various areas, depth, and localization were managed in two burn centers in Bulgaria. Demographic and statistical analyses had been carried out. Topical wound care started with SSD and was changed with Acticoat and Aquacel Ag.

Results: The mean age of the neonates was 17.32 6.7 days. The scalds were the most common mechanism of the injury. The mean TBSA burnt was 7.16% 8.11. The most common areas affected were the face, chest, and upper limbs. Seven sustained superficial partial-thickness burns, ten deep partial-thickness, and two full-thickness injuries. The mean hospital stay was 9.31 7.80 days. The mortality was 5.26%. Hypertrophic scars were observed in 9 babies. The burn wounds fully epithelized in 16 neonates. Only 3 babies were operated on in one-stage procedure—early tangential excision and split-thickness autografts.

Conclusions: Neonatal burns are extremely challenging to the burn team and require special attention. Adequate resuscitation, close monitoring, topical wound care, and skin grafting (when indicated) remain the keynote of treatment.

*P05*

Multiple escharotomy and meshed skin graft for severe burns

**Milosao Bazo** ^1^, Prof. Gjergji Belba ^1^, Prof. Monika Belba ^1^

^1^ University Hospital Center Mother Teresa, Tirana Albania, Tirana, Albania

Aim

To give data for severe burn patients during 2021–2023 and to describe our surgical methods of the treatment of severe burns.

Methods

The study includes patients with severe burns that need surgical wound management.

Results

There were 436 patients with severe burns during the three years in the study, while the mortality rate was 8.4% and 20% of them had burns over 40% TBSA. In the absence of a skin bank, skin substitutes, and cultured keratinocytes, we perform multiple escharotomy, not simple escharectomy, within the first 2–3 days, late escharectomy to eliminate necrosis between the 2nd and 3rd week, and the closure of the wound in several surgical sessions with meshed autograft.

Discussion

Survival in severe burns is achieved with adequate resuscitation during the shock phase, multidisciplinary treatment in sepsis, and local wound intensive treatment and elective surgery (depending on the burning surface, the thickness of the burn, and the region involved). Selective surgery means multiple escharotomies (on the 2nd, and 3rd day), and late escharectomy (between the 2nd and the 3rd week). Afterwards, we have performed a skin graft for functional restoration or a partial-thickness meshed skin graft (for complete wound closure).

Conclusions

Multiple escharotomy helps surgical wound management. Late escharectomy turned out to be a more active procedure. We can save lives with burns to 60% of TBSA, of which 30 to 40% of TBSA was full-thickness.

*P06*

Ex vivo and in vitro burn wound models for studying colonization and testing novel antibacterial treatments

**PhD Bouke Boekema** ^1,2^, Tianne Spreij ^1,3^, Marcel Vlig ^1^, Anouk Elgersma ^1^, Michèle Molendijk ^3^, Dr. Willem van Wamel ^3^, Prof. Dr. Esther Middelkoop ^1,2,4^

^1^ Burn Research Lab, Alliance of Dutch Burn Care, Beverwijk, Netherlands, ^2^ Department of Plastic, Reconstructive & Hand Surgery, Amsterdam University Medical Centre, Amsterdam, Netherlands, ^3^ Medical Microbiology and Infectious Diseases, ErasmusMC, Rotterdam, Netherlands, ^4^ Burn Centre, Red Cross Hospital, Alliance of Dutch Burn Care, Beverwijk, Netherlands

Aim: To investigate the dynamics of wound colonization and to test novel treatments in animal-free burn wound models

Methods: Healthy skin was obtained from adult patients who underwent elective surgery. To construct Full Skin Equivalents and Epidermal Constructs, keratinocytes and fibroblasts, isolated from 0.3 mm split-thickness skin, were cultured in a dermal substitute (Matriderm^®^). For reference, 0.8 mm split-thickness skin samples were cut into squares of 1.5 cm^2^ to create ex vivo burn wound models. The burn wounds were made by a soldering iron (80 °C for 30 s) on the epidermal side without exerting pressure. The models were inoculated with strains of Staphylococcus aureus or Pseudomonas aeruginosa. Various treatments were applied and bacterial survival was determined by plating serial dilutions. Wound healing effects were assessed by immunohistochemistry.

Results: The skin morphogenesis of the Full Skin Equivalents and Epidermal Constructs resembled that of the ex vivo skin and epidermis, respectively. Bacterial survival greatly differed between the strains, skin donors, and model systems that were used. The tested therapeutics showed different profiles in terms of bacterial reduction and wound healing, roughly in the following order: L-Mesitran Soft > silver sulfadiazine > AgNO_3_ > gentamycin > bacteriophages > Medihoney > fusidic acid > untreated.

Conclusions: These animal-free burn wound models greatly support research on skin regeneration and serve as a preclinical platform for testing therapeutic interventions aimed at combatting bacteria and enhancing wound healing. Further development of the models will include the addition of immune cells, which integrate the inflammatory processes in relation to colonization and healing.

*P07*

Advancing Burn Care: Harnessing Hyperbaric Oxygen Therapy for Enhanced Wound Management

**Dr. David Borg** ^1^

^1^ Mater Dei Hospital, Birkirkara, Malta

Aim: This literature review aims to evaluate the efficacy and safety of hyperbaric oxygen therapy (HBOT) as an adjunctive treatment in burn wound management, and to identify optimal protocols for its integration into multidisciplinary care.

Methods: A comprehensive search of electronic databases was conducted to identify the relevant studies published up to [specific date]. The studies investigating the use of HBOT in burn wound management were included. Data extraction focused on study design, patient characteristics, HBOT protocols, outcomes measured, and adverse events reported.

Results: Our analysis revealed a body of evidence supporting the beneficial effects of HBOT in burn wound healing. Studies consistently demonstrated improvements in wound closure rates, reduction in wound depth, and decreased risk of complications such as infections and hypertrophic scarring. Mechanistic studies elucidated the role of HBOT in enhancing tissue oxygenation, modulating inflammatory responses, and promoting angiogenesis and collagen synthesis.

Conclusions: Hyperbaric oxygen therapy shows promise as an adjunctive therapy in burn wound management, offering potential benefits in improving tissue perfusion, accelerating wound healing, and reducing the incidence of complications. However, further research is warranted to optimize treatment protocols, determine patient selection criteria, and elucidate long-term outcomes. The integration of HBOT into multidisciplinary burn care protocols has the potential to enhance patient outcomes and contribute to the advancement of burn management practices.

*P09*

Green burn unit—the pharmacist’s role in environmental sustainability in an interdisciplinary approach

**Marisa Caetano** ^1^, MD Gonçalo Tomé ^2^, Nurse Diana Afonso ^2^, MD Margarida Marques ^2^, MD PhD Luís Cabral ^2^

^1^ Pharmacy Department—CHUC (ULSCoimbra), Coimbra, Portugal, ^2^ Coimbra Burn Unit—CHUC (ULSCoimbra), Coimbra, Portugal

Aim: The identification of activities developed by pharmacists that can reduce the environmental impact of medicines and medical devices in a burn unit.

Methods: Research online for initiatives that can be employed in a burn unit. Analyze daily practices and operations related to the prescription of these products, the type of consumption pattern, and pharmaceutical interventions.

Results: Several initiatives, experiences, and examples were found on the internet. regarding environmental sustainability and daily practice, some critical areas were identified: the operating room (type of anesthetic gasses employed, and material waste) the wound dressing changes (devices that are more easily biodegradable, or that can be used for a longer period of time), intravenous medication, and invasive interventions. Concerning the interventions performed by the pharmacist, one may refer to medication optimization, which encompasses medication reconciliation, medication description, switching from intravenous to oral medication, and personalized therapy, like therapeutic drug monitoring, among others.

Conclusions: The prescription and supply of medicines and medical devices are necessary for managing a severely burned patient. Pharmacists can play a significant role in minimizing the environmental impact of these products. Similar to the management of better patient care with a multidisciplinary team, an environmental stewardship team would also be beneficial to implement several measures for a green burn unit.

*P10*

Effect of early norepinephrine on renal outcomes in critically ill burn patients admitted to the London Regional Burns Service

**Robyn Senior** ^1^, **Dr. Ishani Canisius** ^1^, Jason Tatlock ^1^, Sabri Soussi ^2^, Suveer Singh ^3^

^1^ Chelsea & Westminster Hospital, London, United Kingdom, ^2^ University Health Network, Toronto, Canada, ^3^ Imperial College London, London, United Kingdom

Aim:

To assess whether early exposure to norepinephrine is associated with an increased incidence of acute kidney injury (AKI) and/or the need for renal replacement therapy (RRT) in patients admitted to intensive care following a burn injury.

Methods:

Retrospective observational study. Adult patients admitted to Chelsea & Westminster Burn Intensive Care Unit between May 2020 and January 2024. Independent variables (AKI biomarkers, KDIGO criteria, and RRT), and dependent factors were collected from hospital electronic health records. Descriptive proportions, correlation, and logistic regression were employed. Preliminary single-center data analysis is part of an ongoing multi-center international study.

Results:

A total of 29 consecutive patients were analyzed. Median age 56 years, 48% female, 52% male. In total, 16 developed AKI 1 with a median peak norepinephrine infusion rate of 0.25ug/kg/min vs. 0.09ug/kg/min in those without AKI 1. U = 58 *p* = 0.045. Effect size r = 0.36. Logistic regression analysis (Chi2(1) = 3.88, *p* = 0.049, n = 29). Odds ratio is 36.14. Eight patients received RRT during admission, with a median peak noradrenaline infusion rate of 0.24 ug/Kg/min vs. 0.16 ug/Kg/min in those who did not. U = 54, *p* = 0.153. Effect size r 0.27. (Chi2(1) = 2.84, *p* = 0.092, n = 29).

Conclusions:

A higher norepinephrine rate within 48hrs of burn injury is associated with a significantly increased chance of developing AKI 1. A higher norepinephrine dose did not portend an increased requirement for RRT. The predictors of AKI, triggers for norepinephrine, and implications require further study. International multicentre data collection is ongoing.

*P11*

Advantages of using a nanocellulose-based dressing in facial burns in pediatric patients

**Maria Cantú** ^1^ Zulema Cantú ^1^ Paloma Vázquez ^1^

^1^ Hospital Pediátrico Tacubaya Unidad De Quemados, Benito Juárez, Mexico

Introduction: Facial burns in children are a challenge in management due to the functional and aesthetic implications. Dressings that facilitate their management as well as improve their results will be a great advance.

Objective: To show the benefit of using nanocellulose-based wound dressing in facial burns in children.

Materials and method: Prospective and observational study in patients from the burn unit of the Tacubaya Pediatric Hospital México City, September and October 2023.

Inclusion criteria: pediatric patient, facial burn of less than 72 h of evolution, first and second degree, and mechanism of injury: scald, direct fire, electricity, and deflagration.

Results: During the study period, 7 patients were reviewed, observing greater ease in the management of facial burns, reducing the number of change dressings and sedations, adequate epithelialization at the burn site without the presence of contamination or infection, and good aesthetic and functional results. In some cases, we were able to manage on an outpatient basis.

Conclusions: The bacterial nanocellulose dressing facilitates the management of facial burns, reduces the number of sedations by moderately requiring a dressing change, and promotes the epithelialization of the burn with good aesthetic and functional results.

Keywords: bacterial nanocellulose; wound dressing; pediatric burns

Bibliography: Julia Cattelaens ^1,^*, Laura Turco ^2^, The Impact of a Nanocellulose-Based Wound Dressing in the Management of Thermal Injuries in Children: Results of a Retrospective Evaluation, Life, 2020, Vol 10, page 212.

*P12*

Combination of Hydrosurgery (Versajet™) and Bacterial Nanocellulose (Epicite hydro^®^) for the Management of Complicated Deep Partial Thickness Facial Burns: Case Report

**Dr. Jose Joel Casas Beltran** ^1^, PhD Ives Bernardelli de Mattos ^2^

^1^ Department of Burn Unit, Hermosillo, Mexico, ^2^ Department of Tissue Engineering & Regenerative Medicine (TERM), Würzburg, Germany

Aim:

Demonstrate the safety, clinical use, and advantages of the combination of hydrosurgery and bacterial nanocellulose for complicated facial burns

Methods:

Case report: The case involves a 37-year-old female patient who suffered a facial burn and trauma followed by an explosion and direct fire referred 4 days after the event with the presence of eschar and infection by Acinetobacter baumanii. The treatment consisted of hydrosurgery for scarectomy and debridement, followed by coverage with an acellular dermal substitute composed of bacterial nanocellulose previously immersed in Prontosan^®^ for 30 min, covering all the affected areas.

Results:

The recovery process followed by the treatment showed a visual analog for a pain scale of 1 (VAS), a decrease in the inflammatory process and edema, the absence of infection or complications, complete re-epithelialization at 7 days, and a scar scale assessment score of 1 at 6 months (VSS). The patient required only one surgical procedure and coverage therapy, showing a decreased use of analgesics and a short length of hospital stay.

Conclusions:

This case demonstrates that the combination of technologies for debridement and coverage in non-recent infected burns is safe. The clinical benefits of using bacterial nanocellulose as a dermal substitute are not affected by the presence of complications once the wound bed is prepared with hydrosurgery. It is a potential treatment option for surgeons as it facilitates surgical treatment.

*P17*

Lyophilized and Acellular Fish Dermis Regenerative and Antibacterial Properties in Management of Complicated Traumatic and Surgical Wounds

**Dr. Alfredo Cordova** ^1^, Sammy Shihadeh ^2^, Victoria Young ^3^, Talia Selembo ^1^, Nicole Greenwood ^3^

^1^ Sarasota Memorial Hospital, Sarasota, United States, ^2^ Florida State University College of Medicine, Sarasota, United States, ^3^ Lake Erie College of Osteopathic Medicine, Bradenton, United States

Aim:

Despite repeated excisional debridements and aggressive wound care, burn wounds, traumatic injuries, surgical wounds from abdominal catastrophes, and necrotizing soft tissue infections (NSTIs) may remain heavily colonized. Most skin substitutes are highly sensitive to bacterial colonization and infection. Decellularized and lyophilized fish dermis (DLFD) have been shown in vitro to possess effects decreasing bacterial migration and proliferation acting as a bacterial barrier. Omega-3 contributes to these attributed effects. DLFD may serve with bacterial protection and enhance optimal wound regeneration in preparation for grafting.

Method:

Multiple patients with numerous comorbidities sustaining full-thickness skin defects and complicated wounds with at least heavy bacterial colonization were included. These patients had sustained traumatic and surgical wounds from abdominal sepsis and NSTI, and they underwent prior debridements and local wound care. The application of DLFD and negative pressure wound therapy was then performed. Subsequently, they underwent resurfacing with a split-thickness skin graft (STSG).

Results:

Despite the bacterial colonized environment, complete xenograft incorporation and wound enhancement for grafting were noted within 5 to 14 days. Graft integration and optimal granulation tissue were evidenced in >95% surface area as early as 5 days after product application. No graft loss occurred. Subsequently, STSG revealed nearly 100% graft-take and epithelization within 2 weeks.

Conclusions:

DLFD provides excellent wound coverage of colonized wounds, acts as a bacterial barrier, and enhances the formation of an optimal wound bed for skin grafting. Even though these properties have been observed, we do not advocate using any skin substitute on an infected field. Adequate wound bed preparation is paramount for the success of our patients.

*P21*

Brachial artery perforator flap venous congestion salvage in burn patients: a role for venocutaneous catheterization

Gonçalo Tomé ^1^, **Dmitry Shelepenko** ^1^, José Azevedo ^1^, Inês Catalão ^1^, Miguel Sítima ^1^, Rui Almeida ^1^, Carla Diogo ^1^, Luís Cabral ^1^

^1^ Plastic, Reconstructive Surgery and Burns, Coimbra University Hospital Centre, Coimbra, Portugal

Aims: To evaluate methods to address flap venous congestion, particularly venocutaneous catheterization (VCC), and the potential of brachial artery perforator (BAP) flap for elbow wound coverage in burn patients.

Methods: A two-case report of 50- and 73-years-old male patients, victims of electrical 3rd degree burn and a 3rd-degree acid burn on the upper limbs, submitted to BAP propeller flaps for biceps tendon exposure in the cubital fossa and olecranon exposure, both complicated with venous congestion and, respectively, addressed with VCC and venous supercharge (VS). We additionally conducted a literature review.

Results: Either the VCC of an isolated superficial vein with serial venous drainage for 3 days or VS were key elements for BAP flap salvage due to venous congestion. Discharged after 51 and 18 days, respectively, no infection was observed. However, blood transfusions were necessary. After 36 and 4 months, respectively, the flaps healed evenly without functional impairments. VCC represents a simple, safe, and effective alternative to microsurgical VS, leeches, local subcutaneous heparin injection, or negative pressure therapy for flap venous congestion, which is often difficult to manage. Elbow coverage is similarly challenging, particularly in burn patients. BAP flap has constant perforators, identified by hand-held Doppler, low donor-site complications, good aesthetic, and functional results, more easily used than free flaps for elbow defects in burn patients.

Conclusions: VCC is a simple, relatively safe, alternative solution to perforator flap venous congestion. BAP flap is a relatively straightforward option for elbow coverage.

*P26*

Distribution and Antibiotic Sensitivity of Burn Wound Colonization in Referred Patients at the Burn Care Center, PIMS, Islamabad, Pakistan

**Ali Mujtaba** ^1^

^1^ Pakistan Institute Of Medical Sciences, Islamabad, Pakistan

Background:

Burn injuries represent a significant health challenge worldwide due to their high mortality and disability rates. These wounds often become prone to infection due to compromised immunity and disrupted blood vessels at the injury site. Timely surgical excision, coupled with topical chemotherapy, has notably reduced mortality rates in burn patients.

Objective:

The objective of this study was to determine the prevalence of common bacterial infections among referred patients, with a minimum delay of one week, and to assess their bacteriological susceptibility at the Burn Care Center, PIMS, Islamabad.

Methods:

This retrospective descriptive study was conducted between March 2021 and February 2023. We reviewed wound swab culture results spanning 24 months, primarily from referred patients. The wound specimens were collected using sterile swabs and standard techniques from registered patients, and then analyzed at the microbiological laboratory based on patients’ culture reports.

Results:

A total of 226 culture reports were assessed, comprising 95 (42.04%) male patients and 131 (57.96%) female patients, with a mean age of 21 years (range: 11 months to 65 years). Among these reports, 16 (7.08%) showed no growth. Pseudomonas aeruginosa was identified in 88 patients (38.93%), while Staphylococcus aureus (MRSA) was present in 57 (25.22%), Klebsiella pneumoniae in 36 (15.93%), and a combination of Klebsiella and Pseudomonas in 18 (7.96%) cases, as per the swab culture reports. Escherichia coli was detected in three (1.33%) cases and Enterobacter in two (0.88%) cases. Other bacteria were found in six (2.65%) swab culture reports. Pseudomonas and Klebsiella exhibited maximum susceptibility to Polymyxin B, at 67.04% and 61.11%, respectively, while MRSA showed the highest susceptibility to linezolid, at 89.47%.

Conclusions:

Pseudomonas aeruginosa, Staphylococcus aureus, and Klebsiella pneumoniae were the most prevalent bacteria among the patients referred to our Burn Care Center. The referred burn patients were found to be colonized with multi-drug resistant bacteria compared to the inpatient cases at our center.

*Poster session 3, 17 October 2024, 15:30–16:00*

*P08*

Evaluation of Hyperbaric Oxygen Therapy as an Adjunctive Treatment for Ischemia in Mastectomy Skin Flaps

**Dr. David Borg** ^1^

^1^ Mater Dei Hospital, Birkirkara, Malta

Introduction: Hyperbaric Oxygen Therapy (HBOT) has garnered attention for its potential in wound healing and tissue salvage. This paper critically examines the efficacy of HBOT as an adjunctive treatment for preventing ischemic complications in mastectomy skin flaps.

Aims:

Using the PICO framework, this study aims to evaluate the effectiveness of HBOT in improving surgical outcomes and preventing ischemia in mastectomy skin flap procedures.

Methods:

A comprehensive literature search was conducted, focusing on studies published between 2018 and 2023. Peer-reviewed publications in English involving human subjects were included, while gray literature was excluded. Data were extracted and critically appraised for relevance and quality.

Results:

The analysis of four studies revealed promising outcomes with HBOT, including flap salvage and reduced reoperation rates. However, variations in study designs and patient cohorts underscored the need for more robust evidence through well-designed randomized controlled trials (RCTs).

Conclusions:

While HBOT shows promise as an adjunctive treatment for ischemia in mastectomy skin flaps, the heterogeneity in study methodologies and the absence of standardized treatment protocols highlight the need for further research. Large-scale RCTs with standardized protocols are warranted to provide more definitive recommendations for clinical practice.

*P13*

Bromelain-based enzymatic debridement of facial burns and conservative treatment with medical-grade honey: experience at Hospital Universitario La Paz in Madrid

**Fátima Cerón Molina** ^1^, José Ramón Martínez Méndez ^1^, Maite Serrano Alonso ^1^, Paula Álvarez Hernández ^1^, Fernando De Juan Santos ^1^, Francisco Leyva Rodríguez ^1^

^1^ Hospital Universitario La Paz, Madrid, Spain

Aim:

Managing facial burns presents unique challenges due to the aesthetic and functional importance of this area. This study describes the experience at Hospital Universitario La Paz in Madrid with enzymatic debridement using bromelain (Nexobrid) and conservative treatment with medicinal honey.

Methods:

Patients with second- and third-degree facial burns treated between January 2020 and December 2023 were included. Nexobrid was applied according to standard protocol, emphasizing preparation and application times. Subsequent treatment with medicinal honey was used to promote healing. Indications, usage protocol, aesthetic and functional outcomes, and common sequelae were evaluated.

Results:

Enzymatic debridement with Nexobrid achieved the rapid and selective removal of necrotic tissue in most cases, significantly reducing the need for additional surgical debridement. The subsequent treatment with medicinal honey facilitated rapid healing and reduced secondary infections. Aesthetically, most of the patients reported high satisfaction with the outcomes, and functionally, complete recovery was observed. The most common sequelae were hyperpigmentation and hypertrophic scars.

Conclusions:

The combined protocol of enzymatic debridement with bromelain and conservative treatment with medicinal honey proved effective for managing facial burns, providing good aesthetic and functional outcomes with minimal sequelae. This experience suggests that this strategy could be a valuable addition to the treatment of facial burns in tertiary burn units.

Keywords: facial burns; enzymatic debridement; bromelain; Nexobrid; medicinal honey; aesthetic outcomes; functional outcomes; sequelae.

*P14*

Efficacy of Flaminal^®^ in reducing surgical intervention rates in pediatric deep partial-thickness burns: a retrospective study

**Dr. Margarida Cerqueira** ^1^, Victória Matos ^1^, Inês Pessanha ^1^, Liliana Santos ^1^, Vanda Conceição ^1^

^1^ Unidade Local De Saúde De Coimbra, Coimbra, Portugal

Aim:

The aim of this study is to characterize the impact of introducing Flaminal^®^ in the treatment of deep partial-thickness burns in pediatric patients.

Methods:

A retrospective study was conducted, analyzing pediatric patients treated for deep partial-thickness burns in our center. The patients were divided into two groups: one group where Flaminal^®^ was used as the treatment and another group without its use.

Results:

A total of 290 pediatric patients were included, of which 56.9% (N = 165) were male. The age of the patients ranged from 28 days to 17 years, with a median age of 2 years. The most frequently affected body area was the upper limbs (N = 156; 53.8%). The average burned body surface area (BSA) was 5.9% (SD ± 6.4). Hospitalization was required in 47.2% (N = 137) of the cases. The average time to epithelialization was 25.3 days (SD ± 28.8). In total, 187 patients were treated with Flaminal^®^ (64.5%). The main results highlighted that in the group treated with Flaminal^®^, surgical debridement was necessary in 8.0% of the cases, compared to 35.0% in the group without its use (*p* < 0.001). As for grafting, it was necessary in 4.3% of the patients treated with Flaminal^®^ compared to 28.2% in the group where this agent was not used (*p* < 0.001).

Conclusions:

From this study, the authors conclude that the use of Flaminal^®^ appears to reduce the need for surgical interventions in pediatric burn patients, with no increase in sequelae or description of adverse effects.

Keywords: Flaminal^®^; burn; graft

*P15*

Healing Quality In Outpatients With Deep 2nd-Degree Burn And Use Of Synthetic Nanocellulose Substitute

**Dr. Enrique Antonio Chau Ramos** ^1^, Guillermo Martin Wiegering Cecchi ^2^, Christian Alexander Chau Ramos ^3^

^1^ Clinica Javier Prado, Lima, Peru, ^2^ clinica Javier Prado, Lima, Peru, ^3^ clinica Javier Prado, Lima, Peru

SUMMARY

Objective:

To compare healing between two dermal substitutes: regenerated cellulose and xenograft in patients with deep second-degree burns.

Materials and methods:

A total of 60 cases were evaluated in a private clinic in Lima, Peru, between January 2022 and July 2023. Patients, aged between 1 and 60 years and without comorbidities, were evaluated within the first 24 h after suffering burns from hot liquid. The study was comparative, interventional, analytical, prospective, and longitudinal. Both dermal substitutes were used simultaneously in all the patients, with prior authorization through informed consent.

Results:

At 90 days, healing was evaluated. The synthetic cellulose dermal substitute showed better healing than the xenograft. The indicators evaluated according to the Vancouver Scale (vascularization, pigmentation, flexibility, and height) favored the synthetic cellulose substitute, with less redness and greater elasticity. This study highlights the importance of evaluating the quality of healing in both donor sites using different substitutes.

Conclusions:

The synthetic cellulose substitute is an efficient alternative for the treatment of second-degree burns. It provides better healing in affected areas compared to xenograft. This new option is used to cover the donor areas and promotes a more effective healing process.

Keyword: graft; biological dressings; tissue donors; cicatrization; burn units

*P18*

Management of Necrotizing Soft Tissue Infections with Decellularized Piscis Dermis and Autologous Suspended Skin Cell Transplantation

**Dr. Alfredo Cordova** ^1^, Sammy Shihadeh, Victoria Young, Talia Selembo, Nicole Greenwood

^1^ Sarasota Memorial Hospital, Sarasota, United States

Aim:

Necrotizing soft tissue infections (NSTIs) are common life-threatening conditions that may lead to significant tissue loss and disfiguring wounds. Its management is challenging and carries significant morbidity. Skin substitutes may enhance the development of an optimal wound bed for grafting and provide temporary wound coverage reducing these risks. Decellularized and lyophilized north Atlantic cod dermis have properties in the four stages of wound healing. Subsequent resurfacing with autologous split-thickness skin graft (STSG) and suspended skin cell transplantation (SSCT) may lead to the faster and complete healing of the skin grafts with reduced donor sites.

Methods:

Multiple critically ill and comorbid patients presented in septic shock and multiorgan failure secondary to NSTI including Fournier’s gangrene. Different body parts were involved including the extremities, abdomen, chest, buttocks, and the perineum. The patient’s comorbidities included diabetes, alcoholism, cirrhosis, renal insufficiency, coronary disease, morbid obesity, substance abuse, COPD, etc. Excisional debridement was performed. Subsequently, they were grafted with fish dermis graft and later STSG and SSCT.

Results:

Xenograft integration and optimal granulation tissue were evidenced in >95% of the surface area as early as 5 days after product application. This was considered ideal for resurfacing. Skin coverage with meshed STSG and SSCT revealed nearly 100% skin graft take and epithelization in all cases within 2 weeks.

Conclusions:

Decellularized and lyophilized fish dermis provide excellent wound coverage and enhance the formation of the optimal wound bed for grafting for NSTI patients. Subsequently, autologous suspended cell transplantation reduces the time of healing with smaller donor sites and donor site morbidity.

*P19*

Safety and tolerability of a novel drug with antimicrobial and anti-inflammatory properties assessed for three administration routes.

**Dr. Myles Dakin** ^1^, Prof. Richard Aspinall ^1^, Dr. Tom Kenny ^1^

^1^ Hypo-stream Ltd., Cambridge, United Kingdom

Aim:

Assessing a medicinal composition (PAC) for progression to clinical testing in burns.

Methods:

A PhD program confirmed a composition (PAC-M3) possessing anti-Il-6, anti-TNF-a, bactericidal, and virucidal (including protein-spike degradation) activities. Open-label assessments of safety and tolerability were undertaken:

i. Topical wash and dressing drench to PDDFT burns with confirmed G-ve colonization (n = 46).
ii. Peri-operative wash for surgery (n = 318).
iii. Inhalation of nebulized M3 (n = 18), volunteers.

Patient monitoring: groups ii and iii 24 months post-administration; group i until discharge.

Results:

Group i: No reported adverse effects. Colonization and infection cleared; analysis signaled survival benefit correlated with anti-Il-6 action.

Group ii: No reported adverse effects. Analysis suggested a decreased healing time.

Group iii: Only reported adverse effect is a quickly resolving mild cough.

Conclusions:

The in vitro analysis of PAC-M3 revealed the functional neutralization of Il-6 and TNF-a within fifteen minutes alongside bactericidal and virucidal action. Cell survival studies optimized composition to retain anti-inflammatory and antimicrobial actions, without cellular harm.

The relationship between mortality in burns and elevated Il-6 is uncontentious. Burn site infection is highly correlated to skin graft failure and repeat graft surgery, compounded by drug-resistant species found in up to 50% of burn unit swab samples.

Surgical site infection can result in serious complications, and delayed healing is reported in 15% of surgeries. Drug-resistant species have a negative impact on the risk profile of all surgery.

A well-tolerated aerosolized anti-inflammatory, antimicrobial drug offers potential as a therapeutic for ARDS. This compound warrants further investigation for a range of therapeutic uses.

*P20*

Assessment of the pH alteration in acute burn wounds treated with PolyLactide Membrane versus those treated with silver-based dressing

**Mehmet Demircan** ^1^

^1^ Inonu University, Malatya, Turkey

Aim:

The pH value of wounds is a dynamic factor that can change drastically with therapeutic interventions.

We hereby present our study assessing pH changes in pediatric acute burn wounds treated by polylactic membranes (PLMs) versus silver dressings.

Methodology:

Twenty acute pediatric burn wounds with scalding and 10–20% TBSA were included in this study. The pH assessment was performed using litmus paper testing on days 0,3, 7, and 14. All the wounds were dressed in a similar sequential manner according to their treatment group (PLM or silver). On day 14, the re-epithelialization rate was assessed in both groups.

Results:

The pH value of the fresh acute burn wounds was between 8 and 9 on day 0. On days 3 and 7, the pH value shifted to an average of 7.5 in the PLM group and an average of 5.5 in the silver group. On day 14, the pH of the PLM group was at an average of 7 whereas the silver group had an average pH value of 6.

On day 14, re-epithelization was 100% complete in the PLM group while it was 60% in the silver group. The wounds in the silver dressing group reached 100% re-epithelialization on day 21.

Conclusions:

Our study shows that the application of PLM shifted the pH of the wound to an ideal value for healing (between 7 and 8). The silver dressing on the other hand shifted the pH to acidic.

The release of lactate from PLM may create an ideal pH environment.

*P22*

Pediatric burns and time off school—the impact of early school return on healing time and complications

**Miss Lydia Hiddema** ^1^

^1^ Sheffield Children’s NHS Foundation Trust, Sheffield, United Kingdom

Aim:

Assess complication rates in children returning to school before complete wound healing who have met the set criteria and propose guidelines for school return if no increase in complications, with the goal of reducing the burden of staying home on the patient and family.

Methods:

Online records for included cases of burns referrals between January and June 2023 were reviewed for data on burn, management, school return, and complications. Criteria agreed (e.g., age 5+, maximum unhealed area 1% TBSA) for the trial period of early school return; trial data collection is ongoing.

Results:

First cycle: 8/113 (7.08%) complications; 2/77 (2.60%) with unclear school return (infection, wound breakdown); 2/19 (10.53%) remaining off school (sepsis, graft failure due to PICO failure); 4/17 (23.53%) returning before wound healing. Three sought healthcare late, with associated school return prior to specialist care; all developed infections. One case developed infection + wound dehiscence after returning to school early due to prolonged (95 days) healing time.

Second cycle data collection is underway; thus far no complications identified in the patients returning to school before complete wound healing upon specialist advice.

Conclusions:

Time off school following burns has educational and social impacts on children, and financial burdens on guardians, especially since the cost-of-living crisis. Return to school before specialist care appears to be the biggest risk factor for infection post-burn, potentially contributing to delay in healing. No clear risk increase was seen when advised by a specialist team; therefore, criteria for appropriate school return were implemented, and data are currently being collected to assess the impact on patient outcomes.

*P23*

Mortality estimates of burns in the older adult: A Northern Ireland regional update

**Dr. Hana Humphrey** ^1^, Mr Abid Rashid ^1^, Mr Brendan Fogharty ^1^, Miss Claire Black ^1^, Dr. Amy Doran ^1^

^1^ Regional Burns Unit, Royal Victoria Hospital, Northern Ireland

Aim:

The physiology of older patients sets them apart as a distinct cohort in the management of burns. The aim of this audit was to collate local data encompassing two decades of burn morbidity, mortality, and epidemiology in older patients (>65 years) and provide an update of the region-wide mortality estimated with comparison to previously reported local data (1996–2005, 2010–2015).

Methods:

A retrospective audit of all patients over 65 years admitted with thermal injury to the Regional Northern Ireland Burn Unit and Intensive Care Unit (ICU) from the five-year period (1 January 2019–31 December 2023) was conducted by the review of Electronic Care Records and case notes. The primary outcome measure was mortality. The secondary outcome measures were age, percentage burn total body surface area (TBSA), cause of injury, presence of inhalation injury, admission to ICU, and length of stay.

Results:

A total of 85 patients (50M: 35F) were identified compared with 96 (2010–2015) and 143 (1996–2005), respectively. Gender profiles were comparable. The mean age of admission was 78 years, 75 years, and 76.7 years, respectively. In this cohort, seven patients died from burn injury (14%). Cause of fatal burn: three scald, four flame burn, and two with concomitant inhalation injury. Mean age at death is 80 years. The length of admission prior to death varied (0–41 days). TBSA fatal burn, mean 50% (range 9–80%).

Conclusions:

There is a demonstrable reduction in admissions and mortality in older patients with thermal injury over time due to multifactorial epidemiological and clinical factors. Of note, COVID-19 did not affect mortality.

*P24*

Topical desiccating agent (debrichem): an accessible debridement option for removing biofilm in hard-to-heal wounds.

**Steven Jeffery** ^1^

^1^ Pioneer Wound Clinics, Bentley Pauncefoot, Nr Bromsgrove, United Kingdom

Aim:

A single-center, non-comparative evaluation was undertaken to observe the clinical results achieved when following best practices for the application of debrichem.

Methods:

A total of 21 consecutive patients with complex, non-healing wounds of various aetiologies were studied. Wound size, pain exudate, and the proportion of granulation tissue were determined.

Results:

Over the 4-week follow-up period, there was a decline in the mean percentage of devitalized tissue present on the wounds, reducing from 69% at baseline to 49% at week 4. Most of the devitalized tissue was slough, for which the mean baseline percentage was 63% compared with an endpoint of 49%. Conversely, the mean percentage of granulation tissue increased from 31% at baseline to 51% at week 4. The mean visual analog pain score reported during application was 4/10, where 0 represents no pain. However, general wound-related pain scores improved during the follow-up period, with no scores above two at week 2, compared with five at baseline.

Conclusions:

The results indicate that debrichem is a safe and effective method of debridement that requires minimal training and is single-use.

*P25*

Difficulties in treating severe military burns.

**Olga Kovalenko** ^1^, PhD Anton Kovalenko

^1^ Bogomolets National Medical University, Kyiv, Ukraine, ^2^ Burn clinic, Kyiv, Ukraine

Aim:

To evaluate the effectiveness of a treatment program for advanced military burns with delayed removal of necrotic tissue.

Materials and methods:

A total of 38 patients were treated in the burn department of Kyiv Burn Center during 2021–2022. The patients were delivered from the stages of evacuation from the battlefield. The burn center was the third hospital for these patients.

Results:

The patients entered the third stage 6.3 ± 2.1 (3–10) days after the injury. The average age was 36.6 ± 8.3 (25–52). The lesion area was 52.6 ± 7.9% of TBSA (40–70). The wounds of all the patients were infected with polymorphic trench and hospital microflora. Wet necrotic tissue did not allow the excision of the necrosis immediately after admission. Pseudomonas aeruginosa, Acinetobacter baumannii, and Enterococcus faecium Klebsiella pneumonia were mainly grown in wounds. Microflora was resistant to all antibiotics. The patients underwent local treatment of the wounds with hyperosmolar antibacterial ointments to convert wet necrosis into dry; after drying, the necrosis was removed. Antibacterial therapy was carried out according to sensitivity to antibiotics (two antibiotics at the same time). Autograft was started for 21.5 ± 3.1 days.

Necrosis excision was started 4.9 ± 1.2 days after hospitalization in the burn center and 11.2 ± 2.9 days after the injury. The wounds were temporarily closed with xenograft.

Conclusions:

The number of septic complications increased twice as a result of primary wound infection in the group of severe military burns with 40% TBSA. Mortality increased by 1.5 times. Only hyperosmolar ointments and two antibiotics prescribed at the same time made it possible to stabilize.

